# Psychological Distress during Ovarian Cancer Treatment: Improving Quality by Examining Patient Problems and Advanced Practice Nursing Interventions

**DOI:** 10.1155/2011/351642

**Published:** 2011-08-01

**Authors:** Cynthia Kline O'Sullivan, Kathryn H. Bowles, Sangchoon Jeon, Elizabeth Ercolano, Ruth McCorkle

**Affiliations:** ^1^Yale University School of Nursing, New Haven, CT 0653b-0740, USA; ^2^Department of Nursing, Southern Connecticut State University, New Haven, CT 06515-1330, USA; ^3^University of Pennsylvania School of Nursing, Philadelphia, PA 19104-4217, USA

## Abstract

*Background/Significance*. Ovarian cancer patients are prone to psychological distress. The clinical significance and best practices for distress among this population are poorly understood. *Method*. Secondary analysis of research records from a six month randomized control trial included 32 women with primary ovarian cancer. All received 18 advanced practice nurse (APN) visits over six months. Three sub-samples were determined by distress level (high/low) and mental health service consent for high distress. Demographic, clinical factors, patient problems and APN interventions obtained through content analysis and categorized via the Omaha System were compared. *Results*. Clinically-significant psychiatric conditions were identified in 8/18 (44%) high distress subjects consenting to mental health intervention. High distress subjects who refused mental health intervention had more income and housing problems than the other subjects, received the fewest interventions at baseline, and progressively more throughout the study, exceeding the other sub-samples by study completion. *Conclusions*. Highly-distressed women not psychologically ready to work through emotional consequences of cancer at treatment onset may obtain support from APNs to manage cancer problems as they arise. Additional studies may identify best practices for all highly-distressed women with cancer, particularly those who do not accept mental health services for distress, but suffer from its effects.

## 1. Introduction

Patients with cancer who undergo complex treatments may experience psychological distress, but it is often unrecognized, and if left untreated may contribute to poor health outcomes among patients and their caregivers. Evidence-based Distress Management Guidelines, developed by the National Comprehensive Cancer Network (NCCN) include psychological distress screening for all cancer patients early in the course of treatment, so that mental health treatment plans and referrals may be instituted promptly [1]. The guidelines were developed based on the available evidence, which was primarily among women with breast cancer, but little is known about the guidelines' utility with patients with other types of cancer. Ovarian cancer is more lethal, and typically affects women who are older than those with breast cancer [2]. Psychological distress experienced by women with ovarian cancer tends to be worse among younger women, those recently diagnosed, or with advanced forms of the disease or recurrence [3–5], and distress tends to worsen as cancer progresses [6, 7]. It is not known whether the demographic and clinical factors unique to women with ovarian cancer require special refinements to the Distress Management Guidelines when treating their psychological distress. 

Advanced Practice Nursing (APN) interventions, which incorporate physical, educational, psychological, and care coordination interventions during patients' transitions from hospital discharge through chemotherapy, have been associated with improvements in quality of life among patients with cancer [8, 9], including women with ovarian cancer [10]. Oncology APNs focus on symptom management from cancer and cancer treatments, while APNs specializing in psychiatric care are qualified to perform in-depth mental health assessments and may treat or refer patients to other mental health clinicians for psychological problems. The identification of the specific interventions administered by oncology APNs, including those derived from consultation with psychiatric APNs to women with high psychological distress, is important to understand the trajectory of psychological distress in ovarian cancer, evaluate the utility of the Distress Management Guidelines in clinical practice, and determine best practices in managing mental health needs specific to patients with ovarian cancer. 

The purpose of the current study was to examine in depth the documented problems encountered by women with ovarian cancer soon after surgery, as well as APN interventions performed during a series of clinical encounters within six months following hospital discharge. Screening for psychological distress, a component of the Distress Management Guidelines, provided a unique opportunity to analyze the incorporation of this activity into the treatment plan. In addition, it compared problems and nursing interventions for highly distressed patients who received specialized mental health services provided by psychiatric APNs with those patients in the study with both low and high distress who had not received these services.

## 2. Review of the Literature

Because symptoms of ovarian cancer are often subtle and initially attributed to minor problems, more than 70% of ovarian cancer cases are diagnosed at advanced stages when it has metastasized to the liver, intestines, diaphragm, or lungs [2]. Surgical treatment for ovarian cancer includes total abdominal hysterectomy and bilateral salpingo-oopherectomy, an extensive procedure, which entails a difficult postoperative recovery period followed by aggressive chemotherapy. The ability to tolerate the rigors of surgery and chemotherapy is directly related to patients' comorbidities and functional status at diagnosis [11]. The time when women need to adjust to the physical toll of surgery for ovarian cancer, embark on a difficult course of chemotherapy, and contemplate the existential concerns inherent in life-threatening illness may subject them to undo strain and predispose them to sustain psychological distress and its resultant adverse consequences, including anxiety and depression [12]. 

Little available research has examined the specific physical and psychosocial problems experienced over time among women with ovarian cancer and high psychological distress. Norton and colleagues identified mild or greater depressive symptoms in 55% of a sample of women with varying stages of ovarian cancer on the Beck's Depression Inventory [13], and the association of high numbers of symptoms and heightened distress in ovarian or other gynecological cancers has also been reported [5]. Literature is emerging which identifies clinically significant psychosocial and treatment issues among women with ovarian cancer. For example, women under age 60 have higher depressive symptoms and prefer information on coping techniques than older women with ovarian cancer [14]. The need to maintain normality, especially in social relationships, and the tendency to comfort loved ones distraught over their diagnosis, rather than the reverse, have also been noted [15]. However, few studies outline specific interventions helpful in addressing the psychosocial problems experienced among women with ovarian cancer, including the use of clinical practice guidelines as a way to improve effectiveness in treating mental health issues in cancer. However, several randomized clinical trials support their use in primary care with regard to depression [16–21].

Although busy clinicians in various settings struggle within increasingly contracted timeframes to meet their patients' physical and psychosocial needs, quality cancer care nevertheless requires that it be customized to patients' needs and values, proactive to patients' anticipated needs, provide patient education, and allow patients control over their health care decisions [22, 23]. Several creative initiatives designed to improve care related to these recommendations include incorporating the services of APNs to use a broad array of strategies among diverse samples of patients to deliver care inclusive of the above criteria [24]. In oncology, much of the research on APN effectiveness reflects activities performed in ambulatory or homecare oncology settings. In several well-designed randomized trials, McCorkle's research teams have illustrated the effectiveness of a specialized oncology nursing intervention, delivered through periodic APN visits over the course of several months on physical and psychological outcomes in patients with advanced cancer, including reduced symptom distress, increased independence, and lower hospitalizations among lung cancer patients [8], improved function and mental health in patients with solid tumors [9], and improved survival after cancer surgery [25].

APN activities specified in the above studies targeted pain and symptom management, educated patients and families about cancer, cancer treatment, and self-care, and provided ongoing physical and psychological assessments to promote early recognition and management of clinical problems that might otherwise prompt hospital admissions. To identify key aspects of the intervention most likely responsible for these outcomes, several authors have analyzed the content of the APN interventions through secondary analysis. In one study [25], APN interventions for patients with radical prostatectomy focused predominantly on patient teaching (45%) and psychologically based interventions (20%) [26]. Similarly, activities which focused on teaching and providing psychological support and reassurance were among the most frequent nursing interventions delivered to postsurgical cancer patients [27]. These examples illustrate that much of the interventions effective in improving outcomes for patients with advanced cancer who receive specialized care by APNs may be associated with the influence of psychological, educational, and supportive activities. 

McCorkle and colleagues recently found that a specialized APN intervention program resulted in less uncertainty and better mental health summary measures of quality of life than an attention-control group up to six months after ovarian cancer surgery in a randomized control trial [10]. Highly distressed subjects in the intervention group who consented to an additional psychiatric APN intervention consisting of a mental health evaluation and treatment plan resulted in these women reporting significantly less uncertainty and symptom distress and better physical and mental health summary measures of quality of life over time than the women who did not receive this intervention [10]. These findings highlight the complexity of needs experienced by women with ovarian cancer, as well as the importance of tailoring interventions to patients' specific physical and psychological needs. 

 Identification and classification of patient problems and APN interventions for the purpose of analysis requires the use of a valid and reliable classification system to allow for sufficient detail in both of these parameters, individually as well as in relation to one another. The Omaha System [28] is a research-based, practice and documentation classification system with demonstrated validity and reliability in three decades of prospective [29] and retrospective research [30, 31]. It is one of seven standardized terminology systems recognized by the American Nurses Association [32] and is endorsed by the Health Information Technology Standards Panel [33]. The Alliance of Nursing Informatics (ANI) cites its utility as a point-of-care technology that enables users to capture and represent health data regarding assessment, service, and outcomes [34]. It has demonstrated applicability to diverse patient groups, is relatively easy to use, and is able to capture discrete clinical details throughout the course of care. The system is also computerized, allowing efficient data tabulation and analysis. 

Although research in ovarian cancer has grown substantially over the past decade, longitudinal research examining patient characteristics and treatments for psychological distress, as well as their relationships to one another, are lacking. The current study provided an in-depth examination of the problems experienced by women with ovarian cancer and the discrete APN interventions administered to them over the course of their treatment. These findings contribute to the evolving ovarian cancer literature and present important clinical insight as to the utility of performing distress screening, mental health evaluation, and subsequent mental health treatment plans for distress, in accordance with the NCCN Distress Management Guidelines and in combination with APN processes of care. 

## 3. Research Methodology

This study was a secondary analysis of existing research documents obtained during a previously completed longitudinal study among a cohort of women with newly diagnosed ovarian cancer undergoing standard treatment (surgery and chemotherapy, with or without radiation) [10]. 

### 3.1. Description of Parent Study

**Figure 1:**
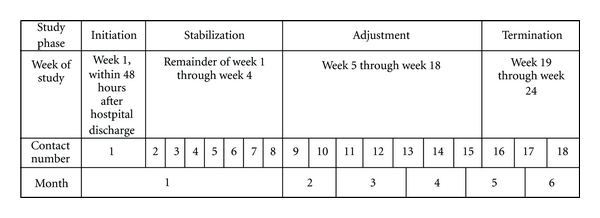
Parent study research phases with corresponding timing and contact numbers.

The parent study was a single-blind prospective randomized control trial, designed to test the hypothesis that women with suspected ovarian cancer who received a specialized nursing intervention program would have greater improvement in quality of life measures over time than women with suspected ovarian cancer in an attention-control group. The parent study's primary investigator was Dr. Ruth McCorkle, and the study was funded through a Grant from the National Institutes of Health, National Institute for Nursing Research, 1R01NR07778. The study included 145 subjects following ovarian cancer surgery. All subjects were screened for psychological distress using the Distress Thermometer (DT) [35] at baseline, prior to hospital discharge. Subjects randomized to the intervention arm (*N* = 73) received 18 contacts by an APN with oncology expertise over six months following hospital discharge. The APN conducted the initial contact and most of the remaining contacts in person, usually at the patient's home or oncology clinic, and the remainder by telephone. The intervention included both standardized and individualized protocols for women following ovarian cancer surgery. The specialized intervention primarily focused on assessment and management of patients' symptoms related to both the cancer and its treatment, while also assisting the patient to gain competence in self-management of these issues over the ensuing months of treatment. The APNs also helped patients navigate the healthcare system and obtain necessary information and resources to improve their experiences and overall quality of life. The intervention spanned six months and was divided into four phases of care: Initiation (baseline assessment, initial symptom management), Stabilization (ongoing assessment and symptom management during chemotherapy), Adjustment (continuing assessment, symptom self-management support and referral), and Termination (referral and support in preparation for discontinuation from study).  Figure 1 illustrates the phases along the six-month care trajectory.

Subjects randomized to the attention control group (*N* = 72) were scheduled to receive eight contacts over six months by a non-nurse research assistant. The first visit occurred in subjects' homes soon after hospital discharge, at which time they received personal instruction and a detailed manual regarding symptom management issues related to cancer treatment. Subjects also received a list of referral personnel, websites, and available volunteer organizations and were encouraged by the research assistants to contact these resources to help them cope with physical or psychosocial concerns experienced during the course of treatment. The resources included names and telephone numbers of area mental health professionals and support groups, as well as the contact information for the principal investigator and project director for additional support and guidance. Subjects were subsequently contacted via telephone by the research assistant six additional times over the course of the six-month study period. 

The major findings of the parent study indicated that the specialized nursing intervention program resulted in less uncertainty and better mental health measures of quality of life than an attention-control intervention up to six months after ovarian cancer surgery. In addition, highly distressed women who received this intervention plus an additional intervention consisting of a mental health evaluation, treatment plan, and ongoing mental health consultation by a psychiatric APN serving in a consultation-liaison nursing role (PCLN) reported significantly less uncertainty and better physical and mental health measures of quality of life over time than women who did not receive the specialized nursing intervention plus the PCLN intervention [10].

### 3.2. Description of Current Study

Subjects for the current study were selected from those who were randomly assigned to the parent study's intervention arm. Included subjects were also required to have the diagnosis of newly diagnosed ovarian cancer, rather than recurrent ovarian cancer or other gynecological cancer diagnoses. All received the standardized specialized APN intervention. This sample was selected to solely study women with ovarian cancer, since its diagnosis is often more insidious, and its prognosis more worrisome than many other gynecological cancers. Because women with recurrent ovarian cancer would likely have more previous knowledge and experience with their disease and treatment than women with newly diagnosed disease, these subjects were excluded to avoid threats to internal validity due to maturation and history effects. 

#### 3.2.1. Sample

**Figure 2:**
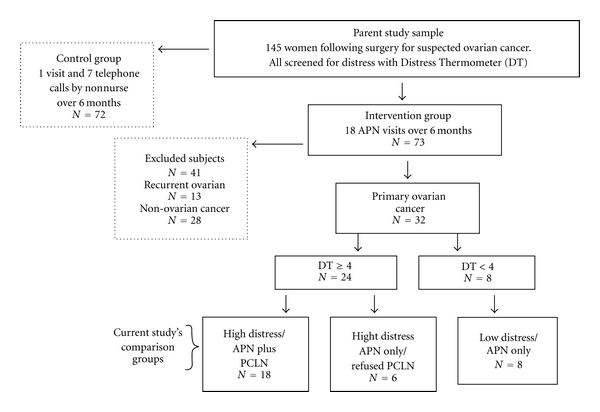
Schematic representation of study's sampling method.

Thirty-two subjects from the parent study sample fit these criteria, twenty-four identified as having heightened baseline measures of distress by scoring at least “4” on the DT [31]. A DT score of 4 or more is considered serious enough to require further evaluation [36]. From the 32 subjects, three subsamples were determined as follows: 18 of the 24 women with high distress agreed to receive the additional evaluation and treatment plan by a psychiatric APN (PCLN). This group constituted one subsample of the current study (the “High Distress/APN + PCLN” subsample). The 14 remaining women with newly diagnosed ovarian cancer did not receive the PCLN intervention; six with high distress who declined the PCLN intervention comprised the second subsample for this study, the “High Distress/APN only/refused PCLN” subsample. Eight subjects scored less than “4” on the DT and were considered to have low distress. These subjects comprised a third subsample, the “Low Distress/APN only” subsample.  Figure 2 illustrates the sampling method.

#### 3.2.2. Instruments

**Distress Thermometer (DT)**

At baseline during the parent study, subjects had completed the DT (Holland, 1996), a rapid screening tool used to identify the presence of distress as endorsed by the NCCN [35]. With this tool, subjects were instructed to mark from a list of thirty-five choices any problems they felt contributed to their distress and indicate their overall level of distress on the tool's visual analog scale. The DT has been found to compare to the Center for Epidemiological Studies-Depression (CES-D) score of 16 in receiver operating characteristic (ROC) curve analysis, with an estimated area ROC curve of.75, suggesting satisfactory sensitivity and specificity [36]. A cut-score of 4 on the DT is reported to have the greatest sensitivity and specificity with the CES-D [33], the Hospital Anxiety and Depression Scale (HADS), and the eighteen item version of the Brief Symptom Inventory (BSI) [37].

**Parent Study Data Collection Forms**

Certain demographic, medical history, cancer-specific, and psychiatric data obtained during the parent study were included for analysis. In addition, work performed by APNs during the parent study and documented on standard pencil and paper forms for each of the 18 contacts provided raw data which was coded using content analysis for the current study. This raw data consisted of checklists and short-answer responses regarding physical, psychological, and social problems commonly experienced by patients with ovarian cancer, reported functional abilities, and health care utilization. It included a nursing care plan, with patient problems, interventions planned and performed, and patient evaluations, with specific notations regarding the types of interventions provided (symptom management and monitoring, counseling and emotional support, education regarding disease, treatments, lifestyle changes, health behaviors, problem-solving, coordination of services and referrals, prescribing of nursing interventions, and direct nursing care) and the focus of the intervention (patient, caregiver, or both). Patient problems and nursing interventions derived through content analysis were entered into a relational database containing the Omaha System codes. Specific logistical data regarding individual visits such as the length, setting (home or clinic), and type of contact (in person versus telephone) were entered into a separate ACCESS database.

PCLN Documentation Forms used for documenting the mental health evaluations and treatment plans consisted of an in-depth mental health assessment using a guided interview format, standard checklists to document common signs and symptoms of major psychiatric illnesses, results of the mental status examination, and the clinical impression regarding psychiatric status. It was one of the tools used to obtain information regarding patients' past psychological history, the PCLNs' clinical impression of the patients' psychological status, and the date, setting, and type (telephone or in person) of the mental health evaluation.

#### 3.2.3. The Omaha System

Patient problems and APN Interventions identified from the parent study records were coded using Omaha System criteria, using established formats as included in *The Omaha System: A Key to Practice, Documentation, and Information Management, 2nd edition *[28], the primary source for use of the Omaha System. The Omaha System consists of a Problem Classification Scheme, an Intervention Scheme, and a Problem Rating Scale for Outcomes [28, 38]. The Problem Classification Scheme consists of four domains: Environmental, Psychosocial, Physiological, and Health-Related Behaviors. Forty-two problems are categorized under one of the four domains and are identified by the signs and symptoms of the problem, the focus of the problem (individual, family or community), and whether the problem is actual, potential, or encompasses the clients' needs for health promotion. 

The Intervention Scheme includes four intervention categories: Health Teaching, Guidance, and Counseling; Treatments and Procedures; Case Management; and Surveillance. Specific nursing interventions further delineate the interventions through the use of 75 “targets,” which describe discreet foci for nursing activities as applied to the four intervention categories [28]. Examples of targets include such items as dressing change/wound care, bowel care, coping skills, and medication administration. Pairing intervention categories with targets specify the intervention, as illustrated in the following examples: *Health Teaching, Guidance and Counseling* for *dressing change/wound care*, *Treatments and Procedures* for *bowel care*, *Case Management* regarding *coping skills*, and *Surveillance* of the patient's *medication administration*. The final Omaha System component, the Problem Rating Scale for Outcomes (PRSO), is used to evaluate patients' knowledge, behavior, and status in relation to outcomes for each problem [38]. The PRSO was not used in this study because there was no available documentation to support assessment of knowledge, behavior, or status for identified problems.

#### 3.2.4. Content Analysis and the Coding Process

Documentation from parent study forms included narrative notes, checklists, and fragments of data which were readily understood by members of the nursing profession, but required classification into a standardized language of problems and interventions for the data to be organized and analyzed. Content analysis is a procedure used to categorize verbal or behavioral data for classification, tabulation, and summarization [39]. The coding process entailed both manifest and latent content analysis of problems and interventions identified. *Manifest content* is content extracted from written, visible components, such as text taken verbatim [40]. *Latent content *is text that provides underlying meaning through its interpretation, requiring the coder to infer from what is written [40]. 

The primary investigator coded the nursing intervention records which met inclusion criteria, coding both patient problems and nursing interventions for each contact according to the criteria defined in the Omaha System literature. Detailed memos regarding coding decisions and their rationale were generated as decisions were made to promote consistency. The entire process was enhanced by ongoing contact with an Omaha System expert who has over 15 years of experience using the system. The expert provided clarification and verification of coding decisions. The results of these discussions were included within the Coding Decision Document.

**Coding Reliability**

Coding reliability was accounted for by using a multistep process involving exercises to evaluate coding stability, reproducibility, and accuracy, as suggested by Krippendorff [39]. Stability was determined by the Principal Investigator through recoding of previously coded phrases several months after the original coding, with the results of the two coding processes compared using percent agreement. A minimum score of 80% was used to provide a measure of extent of agreement beyond chance, as suggested by Kerr [41]. For reproducibility, the entire set of study records from three subjects were recoded by the Omaha System content expert, and these results were compared to the primary investigator's codes using kappa scores, again using 80% as the minimum acceptable score. Both exercises resulted in mean scores of greater than 80%. For accuracy, the Principle Investigator received training from two Omaha System content experts, as well as ongoing supervision and consultation throughout the coding process. During this process, any vague or problematic issues experienced in determining the best coding matches were clarified for standardization and included in a Coding Decision Document, so that future issues would be handled similarly. One of the content experts included the author and codeveloper of the Omaha System, Karen Martin, while the second content expert has conducted extensive nursing research using the Omaha System (Dr. Kathryn Bowles). The expertise of the content experts provided insight into the intent of the given codes, and suggestions for understanding and clarifying entries that were more challenging to identify.

**Data Analysis**

Baseline demographic, medical, cancer-specific, and psychological status were compared overall, and among subjects within each of the subsamples using chi-square for categorical data and ANOVA for continuous data. The Omaha System generated multiple levels of data regarding patient problems and nursing interventions. Discrete data quantifying numbers of patient problems and numbers of interventions were analyzed at increasing levels of complexity, including analysis of these data for the full sample and each subsample overall, per contact, per Omaha System Domain, and per intervention phase. Significance was determined by ANOVA and repeated measures ANOVA as appropriate.

**Human Subjects**

The study was a secondary analysis, and participation did not put subjects at risk for harm or manipulation. Informed consent was previously obtained for the parent study and was subsequently obtained for the current study through expedited review from the Human Subjects Research Review Committee.

## 4. Results

### 4.1. Patient Characteristics

The current sample revealed characteristics similar to other published samples of women with ovarian cancer with respect to age and race, and included subjects who were approximately 60 years old and predominantly white. The majority of the subjects received some college education and had health insurance, but nearly half of the sample reported an annual income of less than $50,000. Most attended religious services, were married, and did not live alone. A comparison of the current sample and subjects from the parent study who were excluded due to having recurrent or nonovarian cancer revealed that the included and excluded subjects were very similar with regard to demographic, medical, cancer-specific, and psychological factors. Of the included subjects, the three subsamples had similar demographic, medical, and cancer-specific factors, but differed significantly with regard to race and education, with the Low Distress/APN only subsample having more nonwhite and fewer college-educated subjects than either of the High Distress subsamples.  Table 1 illustrates the baseline characteristics of the three subsamples.

### 4.2. Omaha System Problems

At baseline, of the 42 available problems, 19 Omaha System Problems were identified among the total sample. Problems identified in at least 30% of the subjects included Mental Health (the most frequent problem), followed by Medication Regimen, Pain, Bowel Function, Digestion/Hydration, and Skin problems. No significant differences in any of the specific problem frequencies were identified among the three subsamples at baseline. For the six-month study period, 26 problems were identified, with Mental Health the most frequent problem, followed by Medication Regimen, Bowel Function, Pain, Digestion/Hydration, Circulation, Skin, Neuromusculo/Skeletal, Sleep and Rest, and Communicable/Infectious Conditions occurring in at least 30% of the total sample. 

Subsamples had similar mean numbers of total problems per contact as well as problems per contact within each Problem Domain. However, when problems per contact were evaluated with regard to the study phases, significant differences emerged at Initiation, with the most Physiological, Health-Related Behavior, and total problems for the Low Distress/APN only subsample, and the fewest Physiological, Health-Related Behavior, and total problems for the High Distress/APN only/Refused PCLN subsample. The latter subsample also had somewhat more Environmental Problems identified in the Stabilization Phase than the other subsamples, but this difference disappeared during the remaining intervention phases. These analyses were limited by small sample sizes, particularly among problems reported within the Environmental Domain (see Table 2). 

Among the eighteen subjects who consented to the mental health evaluation (High Distress/APN plus PCLN subsample), psychiatric diagnoses were identified by the PCLNs in eight subjects. Diagnoses included mood disorders, anxiety disorders, adjustment disorders, and psychiatric disorders due to medical conditions, with two of the subjects found to have more than one disorder. No psychotic disorders were identified (see Table 3). 

### 4.3. Omaha System Interventions

**Figure 3:**
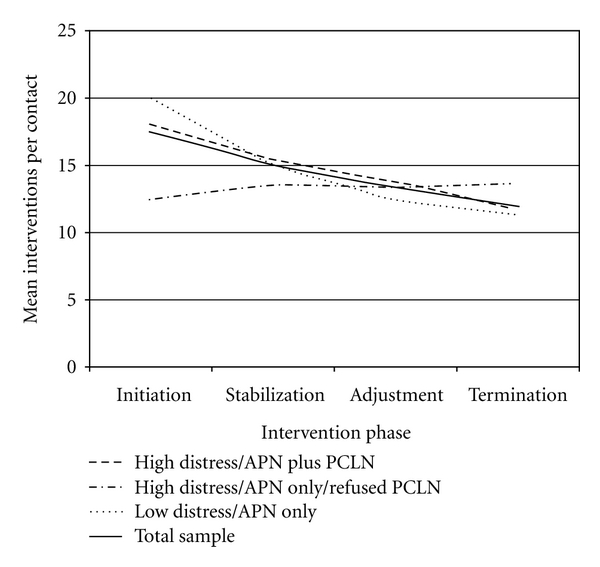
Mean Interventions per contact per intervention phase and subsample: all problems.

Throughout the six-month study, 7,722 interventions were provided to the 32 subjects, which is an average of 241 (±108.6) interventions per subject and 14.0 (±4.6) interventions per contact. Most interventions provided were Surveillance interventions (6,526; 81.46%), followed by Teaching, Guidance, and Counseling interventions (1,196; 15.49%) and Case Management interventions (236; 3.06%). Subsamples had similar numbers of total interventions and interventions per contact, as well as interventions per contact provided within each of the Omaha Intervention Categories. However, when evaluated with respect to when they were administered per the study's intervention phases, the Low Distress/APN only subsample appeared to receive the most interventions per contact at Initiation (baseline), but the least for the remainder of the study period, in comparison to the other (High Distress) subsamples. In contrast, the High Distress/APN only/Refused PCLN subsample appeared to receive the least interventions at Initiation, but gradually more interventions per contact throughout the six-month study period, receiving the most interventions per contact during the study's final (Termination) phase (see Figure 3). No differences were noted in numbers of contacts, length of the contacts, or whether the contacts were delivered in-person or by telephone among the three subsamples.

### 4.4. Problems and Interventions

Examination of the interventions provided with respect to the four Problem Domains revealed several interesting findings. Interventions administered for Environmental Problems were significantly higher for the High Distress/APN only/Refused PCLN subsample than the other two subsamples, although this finding occurred among a very small sample. In addition, differences in numbers of interventions per contact for Psychosocial Problems approached significance, with the High Distress/APN only/Refused PCLN subsample appearing to receive the fewest in comparison to the other two subsamples. A larger sample may provide clarity as to the significance of this observation (see Table 4). 

**Figure 4:**
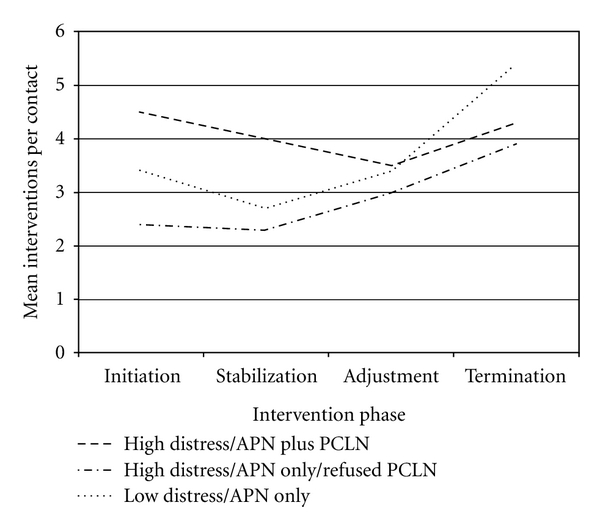
Mean interventions per contact per intervention phase for each subsample: psychosocial problems.

**Figure 5:**
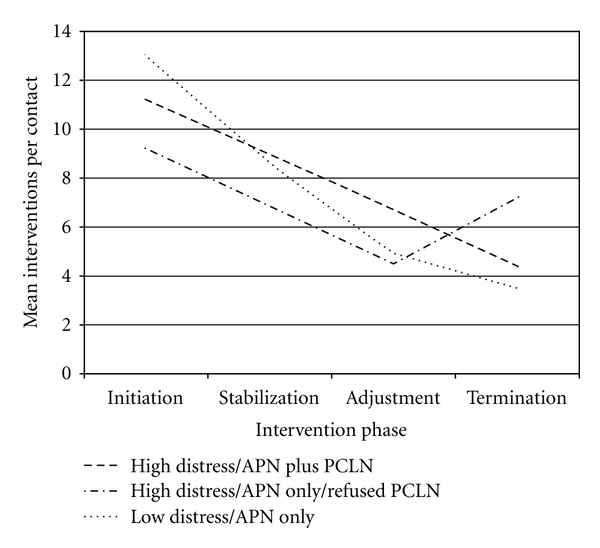
Mean interventions per contact per intervention phase for each subsample: physiological problems.

**Figure 6:**
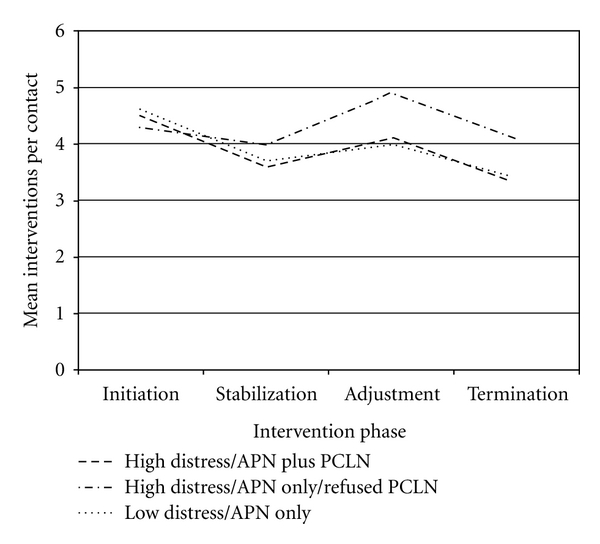
Mean interventions per contact per intervention phase for each subsample: health related behavior problems.

Along the study trajectory, intervention pattern differences were observed relative to numbers of interventions per contact provided within each Problem Domain. In particular, the High Distress/APN plus PCLN subsample initially received the most interventions for Psychosocial Domain Problems than the other two subsamples, but this number remained relatively constant throughout the study period. In contrast, the High Distress/APN only/Refused subsample, and Low Distress/APN only subsample appeared to receive increasingly more interventions for Psychosocial Problems through the end of the study, with this phenomenon markedly apparent for the Low Distress/APN only subsample (see Figure 4). Interventions for Physiological Problems were highest at baseline among the Low Distress/APN only subsample, and this subsample as well as the High Distress/APN plus PCLN subsample received a steady reduction in interventions per contact for Physiological Problems as the study progressed. In contrast, the High Distress/APN only/Refused PCLN subsample did not experience a similar reduction in interventions for Physiological Problems as the study progressed, receiving the most by the Termination Phase (see Figure 5). In addition, interventions for Health-Related Behavior Domain Problems remained relatively constant for all three subsamples as the study progressed, but patterns may indicate a trend of diminishing numbers of interventions along the study trajectory provided to the High Distress/APN plus PCLN and Low Distress/APN only subsamples. A similar pattern was not evident for the High Distress/APN only/Refused PCLN subsample, who received a relatively constant number throughout the study (see Figure 6).

### 4.5. Limitations

The first limitations were related to the study's use of data by secondary analysis of previously-collected data. Since the data had been designed for a different purpose, they required content analysis to quantify them into discrete units for comparison using the Omaha System. Retrospectively categorizing existing data did not allow for further clarification in several instances where, as they were described, signs and symptoms were not able to be straightforwardly coded into Omaha System Problems. This issue highlights a semantic limitation with the Omaha System, which was particularly evident in trying to determine how to classify the symptom “fatigue.” Since fatigue was associated with several Omaha Problems, including Sleep and Rest, Circulation, Digestion/Hydration, and Medication Regimen (when fatigue was considered a chemotherapy side effect), the primary investigator needed to identify the suspected source of the fatigue to correctly identify it as the symptom indicative of a particular Omaha Problem. This was sometimes problematic, because this detail of information was not always evident from the research records. It was therefore possible that errors were made in identifying the correct Omaha Problem associated with fatigue for those patients who experienced this symptom. 

The final limitations were related to the study's sampling. The DT, one of the primary independent variables used to determine subsample assignment, has not undergone substantial cross-cultural validation, and therefore its ability to measure distress among subjects of various ethnicities and levels of education is not known. The current sample was relatively small, with limited diversity. Assignment of subjects into the three subsamples based on subjects' DT scores, as well as their consent for the PCLN intervention, resulted in the subsample with low distress (Low Distress/APN only) being the only subsample with nonwhite subjects. It was also the subsample with the fewest college-educated subjects. It is also not known whether additional factors not explored prior to the analysis may have been associated with these subjects' decision to decline the PCLN intervention. In order to reduce the artificial effect potentially introduced by dissimilar group characteristics that may have been associated with reasons to accept or decline the PCLN referral, a more extensive exploration of baseline and situational factors would have been needed. In addition, a larger and more diverse sample would have provided more reliable evidence as to whether the factors education or race may have influenced the degree of psychological distress or the propensity to decline or accept PCLN care. 

Finally, the sample was derived from a larger sample of women from a single comprehensive cancer center who were being treated for ovarian cancer. The findings therefore are limited in their ability to be generalized to other samples of women with ovarian cancer.

## 5. Discussion

Much of the research on ovarian cancer, including the current study, has been conducted on women who were white and generally older than age 55. However, the significant finding associating nonwhite race and lower education levels among the current study's Low Distress/APN only subsample begs for exploration of the influence of race and education on psychological distress among women with cancer, using larger more racially diverse studies. Further, although the study's results support previous research identifying various symptoms as prevalent among women with ovarian cancer, the novel use of the Omaha System identified unique issues which were not typically explored among this population of women. Specifically, the High Distress/APN only/Refused PCLN subsample may have been less forthcoming with problems and may also have been more distressed due to Environmental Problems such as issues with Income and Residence than either the High Distress/APN plus PCLN or the Low Distress/APN only subsample. Although such environmental issues are not typically associated with ovarian cancer, in the context of difficult ovarian cancer treatment, they may have contributed to already high distress levels. Accurate examination of the unpleasant symptoms and problems experienced by women undergoing ovarian cancer treatment should also explore additional sources of their distress so that appropriately targeted treatments may be initiated. 

One-fourth of the women with high distress refused PCLN care; however, this ratio was better than has been reported by previous studies among cancer patients [42]. The therapeutic relationship established between the APNs and their assigned subjects may have influenced many of the distressed women to accept mental health care. Since improvements in distress among cancer patients has been positively linked to better adherence to cancer therapies, it may be especially important to provide a mechanism for certain patients to establish such therapeutic relationships as a component of their chemotherapy plans, either within the clinic setting, or perhaps as a homecare adjunct to chemotherapy. 

The use of the Distress Thermometer resulted in 24 of 32 subjects reporting high distress. This screening mechanism prompted mental health evaluations to be performed for eighteen women, which revealed that eight of the women were suffering from clinically significant psychiatric conditions, while ten of the women were evaluated as not having clinically significant psychiatric conditions. The most frequent conditions identified included anxiety, mood disorders (depression), adjustment disorders, and psychiatric disorders due to medical conditions. In this manner, the use of the DT in clinical practice, as endorsed by the NCCN guidelines, may be seen as effective in identifying and treating potentially serious psychiatric conditions among cancer patients. Since the DT was easy to use and has shown good reliability with the CES-D and similar measure of psychological distress, it may be advantageous for patients to complete the DT prior to every chemotherapy session, rather than at a single time at the onset of treatment, as occurred in the current study. More frequent monitoring of distress could improve the accuracy of psychiatric problem identification by offering opportunities to compare previous ratings, while also potentially targeting psychiatric evaluation and treatment for those who need it the most. 

 The Omaha System offered a unique method of capturing the broad array of patient problems within all four potential Problem Domains without simply identifying symptoms or targeting a single area of clinical concern among the universe of possibilities. The study findings show the complexity of the ovarian cancer patients' needs, the intensity of nursing care, and the value of a classification system to capture that description. The Omaha System also ensured a high degree of problem specificity by requiring that each active problem be counted only when clearly linked with at least one Omaha System-defined sign or symptom. Many of the Omaha System Problems found closely resembled problems identified among other samples of women with ovarian cancer using different measurement systems. However, the broad range of problems identified enhanced this body of knowledge by also identifying the effects of nontreatment issues sometimes experienced by women with ovarian cancer that may contribute to psychological distress. A major semantic problem occurred with respect to the issue of fatigue and may require a more in-depth examination of the Omaha System's ability to capture in detail the etiology and clinical significance of this problem. 

Interventions identified using the Omaha System were classified into Surveillance, Teaching, Guidance, and Counseling, and Case Management interventions, with Surveillance comprising the largest category. This finding underscored the importance of careful monitoring during the postoperative phase. Although the three subsamples received relatively similar interventions within the Intervention Categories throughout study period, by the end of the six-month period, the High Distress/APN only/Refused PCLN subjects received more interventions overall than subjects in the other two samples. This finding also points to the possibility that this highly distressed subsample may not have initially been ready to discuss psychological issues and may have required additional time to feel comfortable disclosing sensitive information to the APNs. The six-month study period was only beginning to provide the opportunity for them to develop therapeutic relationships sufficiently meaningful to allow for such disclosure. This finding suggested that a six-month time period may be insufficient to allow some patients, even highly distressed ones, to accept certain interventions, but longer-term relationships among patients and clinicians may enhance this ability. 

### 5.1. Implications for Research

Based on the current study's significant findings and methodological limitations, the following suggestions for future research are presented. First, the DT requires additional testing for reliability, validity, and stability among a racially diverse sample with different levels of education and cancer types. Such testing should also include sensitivity and specificity testing to reliably evaluate its use as a screening tool throughout the cancer treatment process in order to fully support its universal usage per NCCN Distress Management Guidelines. 

Second, although the Omaha System is commonly used in practice within the homecare and other settings, more studies using the Omaha System exclusively among cancer patients may provide evidence as to the unique nature of problems experienced by them. One area in need of careful evaluation concerns the issue of fatigue among cancer patients. The Omaha System may offer a viable tool to uncover contributory and mediating factors associated with this elusive problem. In addition, semantic study of this problem for clarity in categorizing it according to Omaha System criteria is in order to improve standardization in documentation.

Third, the longitudinal nature of the current study provided an opportunity for the investigator to examine linkages between patient problems and APN interventions, while incorporating systems characteristics such as the timing of APN contacts and the use of the DT. Further studies which link patient problems, nursing interventions, and outcomes are essential in order for nurses to refine their practice through the merits of evidence. This need is especially important as populations become more diverse and complex, and as the shrinking nursing workforce struggles to meet patients' needs for quality health care. Although secondary analysis was an inexpensive and convenient method for designing and completing this study, future studies among patients with cancer, using the Omaha System in a prospective manner may prove more accurate in correctly identifying Omaha Problems related to specific symptoms and would eliminate the need for content analysis to categorize the data. 

In addition to the Problem and Nursing Intervention Schemes, the Omaha System provides the opportunity to utilize the Problems Rating Scale for Outcomes Scheme, which would be helpful in determining linkages between patient problems, nursing interventions, and patient outcomes. This scheme would enable evaluation of changes within three subscales of patient conditions in relation to specific Omaha Problems: knowledge (patient's understanding about a Problem), behaviors (patient's actions/responses in relation to a Problem), and status (wellness or illness in relation to a Problem) using a five-point rating scale for each subscale. 

Finally, it is possible that extraneous factors not identified may have influenced patients' inclinations to accept or decline PCLN or other mental health referrals. The analysis plan for future studies will need to adjust for these factors, and the results from these studies will need to be evaluated within the context of how these factors versus the intervention alone may account for the results so that the possibility of an artificial effect imposed by these factors is minimized.

### 5.2. Implications for Practice

The current study highlighted clinical outcomes resulting from distress screening for women in active treatment for ovarian cancer. The DT isolated unique phenomena among women who reported varying levels of distress at baseline, which may be helpful to clinicians who care for this cancer population. Women with low distress (the Low Distress/APN only subsample) appeared to be very open to communicating their needs and concerns, were able to articulate their needs to APNs, and became active participants in achieving their health goals, as evidenced by the clear reduction in their problems and interventions as the study period progressed. Those with high distress who were willing to receive services to treat this distress (the High Distress/APN plus PCLN subsample) also appeared to receive valuable assistance in caring for their health during the cancer treatment period through interactions with oncology and psychiatric APNs. Several of these women were identified to have psychiatric conditions worthy of further treatment and were referred appropriately. However, the High Distress/APN only/Refused PCLN subsample presented challenges unique to this subsample. These women may have experienced more Environmental Problems contributing to their distress; therefore, clinical settings need to provide ample opportunities for women to receive assistance in meeting financial, residence, and employment needs, which although not directly related to their disease process, may seriously degrade quality of life during already challenging health events. Clinicians need to be keenly aware of such patients and interact with them with particular sensitivity through continued support and gentle, repeated reminders of how they may be helped. 

The DT was a simple screening tool which identified 24 patients in distress at baseline, with eight evaluated as needing further mental health treatment. The NCCN guideline suggests serial DT screenings to be useful for clinicians to use at baseline and throughout the treatment process, so that areas of distress may be identified and addressed promptly [1]. This would be particularly helpful among women who may be reticent to disclose such problems in conversation, but may feel comfortable completing the DT. For these women, the DT in combination with astute, compassionate clinical assessments during oncology visits may provide the best opportunities to uncover clinically-significant psychological distress.

### 5.3. Implications for Policy

Key elements of quality care, including those providing psychological support services and compassionate care to individuals with cancer, are recognized as essential areas in need of improvement [23, 43]. The recently passed Patient Protection and Affordable Care Act (PPACA) increases funding for general care nurses as well as APNs, with the anticipated outcome being to expand the nursing workforce overall [44]. An area of particular promise is a grant program to fund innovative safety-net programs, such as nurse-managed clinics. Although initially focused on primary care, these safety net programs may also include care for patients who may not be acutely ill, but require management of chronic conditions or support during times of transition (such as from hospital to home). The chronic nature of many types of cancer, including ovarian cancer, which is often characterized by bouts of exacerbations of symptoms over the course of months or years, may be ideally suited for this model of care. Further definition of the APN role in ensuring effective psychosocial care, including teaching, guidance, counseling, case management, and appropriate surveillance is essential at this time in order for these services to be recognized as worthy of reimbursement. 

## 6. Conclusion

The methodological strengths of the Omaha System coupled with the unique opportunities afforded by frequent clinical encounters provided important details about the range of patient problems and APN interventions for women after ovarian cancer surgery not previously described. This study provided extensive information about the specific problems experienced by these vulnerable women as they weathered the course of treatment. It also explored the relationship between documented problems and APN interventions in this sample, including those prompted by PCLN evaluation, treatment, and referral for high distress. Information gained from these descriptions provides evidence useful in examining the clinical processes resulting from screening and initiating a guideline-based clinical plan for psychological distress when experienced by women after surgical treatment for ovarian cancer. Such information is essential for establishing the effectiveness of the current NCCN Distress Guidelines, so that they are most instructive to clinicians who care for women with ovarian cancer in oncology and homecare settings. Promoting the guidelines' utility through appropriate translation methods may facilitate their adoption by clinicians and may support their full integration into the healthcare system through institutional policy reforms. Such enhancements address the priorities endorsed by the IOM and NCCN in relation to health care quality. APNs may provide a critical link in identifying cancer patients in distress, assisting patients to cope with the distress, and referring them appropriately to minimize its adverse effects.

## Figures and Tables

**Table 1:** Baseline demographic characteristics of the sample.

Baseline demographic factors	Total Sample (*N* = 32)	High Distress/APN plus PCLN (*N* = 18)	High Distress/APN only/Refused PCLN (*N* = 6)	Low Distress/APN only (*N* = 8)	Significance between Subsamples* (*P* < .05)
Age mean (±SD)	60.16 (±13.02)	60.31 (±6.94)	58.35 (±5.50)	54.61 (±12.47)	.295

	*N* (%)	*N* (%)	*N* (%)	*N* (%)	—

Race White Not white	30 (93.8) 2 (6.2)	18 (100) 0	6 (100) 0	6 (75) 2 (25)	**.041**

Living situation With someone Lives alone	22 (68.7) 10 (31.3)	15 (83.3) 3 (16.7)	6 (100) 0	5 (62.5) 3 (37.5)	.194

Marital status Married/partnered Not married	23 (71.9) 9 (28.1)	14 (77.8) 4 (22.2)	4 (66.7) 2 (33.3)	5 (62.5) 3 (37.5)	.691

Education ≤High school or technical College or more	7 (21.9) 25 (78.1)	1 (5.6) 17 (94.4)	2 (33.3) 4 (66.6)	4 (50) 4 (50)	**.031**

Presently employed Yes No	16 (50) 16 (50)	8 (44.4) 10 (55.6)	4 (66.7) 2 (33.3)	4 (50) 4 (50)	.798

Annual income <$50,000 $50,000 or more	17 (48.6) 18 (51.4)	4 (26.7) 11 (73.3)	3 (42.9) 4 (57.1)	1 (20) 5 (80)	.565

Religious service attendance Never Occasional Regular	7 (21.9) 11 (34.4) 14 (43.8)	4 (22.2) 5 (27.8) 9 (50.0)	1 (16.7) 1 (16.7) 4 (66.7)	2 (25) 5 (62.5) 1 (12.5)	.249

Health insurance Yes No	30 (93.8) 2 (6.2)	18 (100) 0	5 (83.3) 1 (16.7)	7 (87.5) 1 (12.5)	.241

*Significance determined by chi-square analysis, with the exception of age, which was determined by ANOVA.

**Table 2:** Mean total problems per contact and within each Omaha System Problem Domain, by intervention phase and subsample.

Mean problems per contact per intervention phase and domain				
Intervention phase	High Distress/APN plus PCLN *N* = 18	High Distress/APN only/Refused PCLN *N* = 6	Low Distress/APN only *N* = 8	Significance (*P* < .05)*
Problems/contact: all domains				
Mean (standard deviation)				

Initiation	5.11 (0.28)	3.67 (0.49)	6.87 (0.42)	**<.0001**
Stabilization	1.05 (0.28)	1.21 (0.49)	1.42 (0.42)	.7708
Adjustment	1.27 (0.28)	1.12 (0.49)	1.12 (0.42)	.9402
Termination	2.13 (0.29)	2.18 (0.49)	2.04 (0.42)	.9772

Problems/contact: environmental domain				
Mean (standard deviation)				

Initiation	0.00 (0.01)	0.00 (0.02)	0.00 (0.01)	1.0000
Stabilization	0.00 (0.01)	0.04 (0.02)	0.00 (0.01)	.0994
Adjustment	0.01 (0.01)	0.03 (0.02)	0.01 (0.01)	.5169
Termination	0.02 (0.01)	0.02 (0.02)	0.00 (0.01)	.5636

Problems/contact: psychological domain				
Mean (standard deviation)				

Initiation	1.00 (.07)	0.83 (0.12)	1.12 (0.10)	.1854
Stabilization	0.15 (0.07)	0.23 (0.12)	0.23 (0.10)	.7772
Adjustment	0.22 (0.07)	0.27 (0.12)	0.20 (0.10)	.9101
Termination	0.42 (0.07)	0.39 (0.12)	0.50 (0.10)	.7772

Problems/contact: physiological domain				
Mean (standard deviation)				

Initiation	3.00 (0.24)	2.17 (0.41)	4.50 (0.36)	**.0001**
Stabilization	0.65 (0.24)	0.69 (0.41)	0.78 (0.36)	.9574
Adjustment	0.76 (0.24)	0.53 (0.41)	0.56 (0.36)	.8433
Termination	1.19 (0.24)	1.23 (0.44)	0.99 (0.36)	.8788

Problems/contact: health related behavior domain				
Mean (standard deviation)				

Initiation	1.11 (0.10)	0.67 (0.17)	1.25 (0.15)	**.0305**
Stabilization	0.24 (0.10)	0.25 (0.17)	0.40 (0.15)	.6389
Adjustment	0.28 (0.10)	0.29 (0.17)	0.34 (0.15)	.9494
Termination	0.49 (0.10)	0.53 (0.18)	0.55 (0.15)	.9444

*Significance determined by repeated measures ANOVA mixed effect model, SAS version 9.1.

**Table 3:** Results of mental health evaluation by PCLN.

*N* = 18	Impressions noted by PCLN indicating suspected psychiatric diagnoses	DT	Prior psychiatric history	DSM-IV Axis I major diagnostic categories				
				Mood disorder	Anxiety disorder	Adjustment disorder	Psychiatric disorder due to medical condition	No suspected psychiatric diagnosis
1	Adjustment disorder, generalized anxiety	10				x		

1	Depression related to recent cancer diagnosis.	10		x				

1	Adjustment disorder w/mixed disturbance of mood/sleep problem	10				x		

1	Dysthymic disorder. Psychological disorder (depression + anxiety) due to medical condition. Adjustment disorder with depression/anxiety, r/o major depression	10		x		x	x	

1	History of depression, current presentation has large anxiety component	8	x	x				

1	History of adjustment disorder w/depressed mood. No current diagnosis	8	x					x

1	OCD traits. Generalized anxiety disorder	7.5			x			

1	Generalized anxiety disorder, Adjustment disorder with anxiety- now resolved, Obsessive compulsive disorder traits.	4.5			x	x		

1	Generalized anxiety disorder, rule out major depression, recurrent	4	x	x	x			

9	No diagnosis	Range 4–8.5						x

**Table 4:** Mean interventions per contact per domain.

Interventions per contact within each domain	Total sample *N* = 32	High Distress/APN plus PCLN *N* = 18	High Distress/APN only/Refused PCLN *N* = 6	Low Distress/APN only *N* = 8	Significance between Subsamples*
Environmental domain mean (±SD)	.47 (±.66)	.26 (±.32)	1.19 (±.87)	.21 (±.39)	**.001**
Psychosocial domain mean (±SD)	1.76 (±2.03)	1.90 (±2.10)	1.37 (±1.54)	1.73 (±2.16)	.090
Physical domainmean (±SD)	3.66 (±4.61)	3.79 (±4.57)	3.11 (±4.19)	3.75 (±4.99)	.451
Health-related behavior domain mean (±SD)	1.83 (±2.23)	1.77 (±2.18)	1.97 (±2.17)	1.84 (±2.40)	.768

*Significance determined by ANOVA.
